# Comparison of qPCR and chromogenic culture methods for rapid detection of group B streptococcus colonization in Vietnamese pregnant women

**DOI:** 10.1016/j.plabm.2024.e00435

**Published:** 2024-10-15

**Authors:** Manh-Tuan Ha, Huyen Tran-Thi-Bich, Thao Bui-Thi-Kim, My-Linh Nguyen-Thi, Thanh Vu-Tri, Thuy-Duong Ho-Huynh, Tuan-Anh Nguyen

**Affiliations:** a University of Medicine and Pharmacy at Ho Chi Minh City, Ho Chi Minh City, Viet Nam; b University Medical Center Ho Chi Minh City - Branch 2, Ho Chi Minh City, Viet Nam; c Khoa Thuong Biotechnology Company, Ho Chi Minh City, Viet Nam; d Thu Duc Region General Hospital, Thu Duc City, Viet Nam; e Molecular Biomedical Center, University Medical Center Ho Chi Minh City, Ho Chi Minh City, Viet Nam

**Keywords:** Direct real-time PCR, Diagnostic value, Early-onset neonatal infection, intrapartum antibiotic prophylaxis, *Streptococcus* group B

## Abstract

**Introduction:**

Neonatal infections can rapidly become severe, with delays in treatment often proving fatal. *Streptococcus agalactiae* (Group B *Streptococcus*, GBS) is a common cause, typically transmitted from colonized pregnant women to neonates during childbirth. In Vietnam, routine prenatal care lacks standardized GBS screening protocols. This study aims to compare enhanced qPCR methods with the culture method, evaluate the diagnostic accuracy of these qPCR procedures, and assess the frequency of GBS infection in pregnant Vietnamese women during their final trimester.

**Materials and methods:**

Pregnant women aged 35 weeks gestation or more were recruited. Rectovaginal swabs were collected and analyzed for GBS using chromogenic culture, a commercial real-time PCR kit, and in-house real-time PCR assays targeting the *cfb* and *sip* genes. Clinical diagnostic values were calculated, and GBS prevalence was determined.

**Results:**

The study included 259 pregnant women with a mean age of 30.2 ± 5.0 years. Of these, 96.6 % had gestational ages of 37 weeks or more at delivery. The sensitivity, specificity, positive predictive value, negative predictive value, and accuracy of the *cfb*-based and sip-based qPCR assays were 94.1/92.7, 99.0/99.5, 97.1/98.5, 97.8/97.3, and 97.6 %, respectively. The Kappa values were excellent (0.940 and 0.939), with results available in under 2 h. GBS prevalence was 24.7 % and 25.5 % by *cfb*-based and *sip*-based qPCR assays, aligning with the culture method (25.5 %).

**Conclusions:**

Both direct real-time PCR assays demonstrated high accuracy and were comparable to chromogenic culture in diagnosing GBS. A significant prevalence of GBS colonization was found among Vietnamese pregnant women in their final trimester.

## 1 Introduction

Infection is the main factor in neonatal morbidity [1] and is one of the leading causes of neonatal deaths in Vietnam (2019), accounting for 6 % of all cases (www.healthynewbornnetwork.org/country/vietnam). The neonatal mortality rate is as low as 4.6 %, but a high incidence of infectious diseases has been reported in a Vietnamese provincial hospital [1]. Although the prevalence of neonatal mortality in Vietnam has constantly decreased since 2002, with fewer than 20 000 cases in 2017, infectious diseases, such as acute respiratory infection and sepsis, are the principal sources of neonatal mortality, in addition to birth asphyxia, congenital disorders, and prematurity [2]. Despite being treatable, neonatal infection can be fatal if detected late and treatment is not given early.

Group B streptococcus (GBS), or *Streptococcus agalactiae,* is one of the critical causative pathogens leading to neonatal infections [3,4]. It is a commensal bacterium that resides in the human gastrointestinal tract and urogenital organs, with a prevalence of 30 % in healthy adults [5] and up to 36.3 % of pregnant women [6]. Pregnant women colonized by GBS might vertically transfer the bacteria to infants at delivery, resulting in early-onset sepsis in neonates within 0–6 days of birth [3,7]. Infected neonates might develop bacteraemia, pneumonia, and bacterial meningitis and lose their lives if not detected early and treated appropriately [8]. Moreover, maternal GBS screening and intrapartum antibiotic prophylaxis led to a 70 % reduction in the prevalence of early-onset GBS infection [9].

Guidelines from the Centers for Disease Control and Prevention (CDC) [10], the American College of Obstetricians and Gynecologists (ACOG) [11], the American Academy of Pediatrics (AAP) [12], and the American Society for Microbiology (ASM) [6,13,14] recommend intrapartum antibiotic prophylaxis as the most effective way for GBS-positive pregnant women to reduce the incidence of early-onset neonatal infection. The clinical risk assessment tool and GBS screening tests used to evaluate the GBS status of pregnant women during the last three months of pregnancy allow for determining whether to use antibiotics during induction [15]. However, routine screening for GBS during pregnancy prevents more cases of early-onset disease than the risk-based approach [16]. Moreover, according to the latest recommendations from the ACOG, determining GBS infection of the genitourinary and gastrointestinal tract at any time during gestation indicates heavy maternal rectovaginal colonization. This demonstrates the necessity for intrapartum antibiotic prophylaxis without needing a successive GBS screening rectovaginal culture at 36 0/7–37 6/7 weeks of pregnancy [14]. In the case of pregnant women who are not screened for GBS colonization during pregnancy, examination for GBS infection at the time of delivery and the use of antibiotic prophylaxis are vital for newborn infection prevention [17].

In Vietnam, there are no public health regulations for the regular standardization of GBS screening for pregnant women during the prenatal period. Notwithstanding, some large hospitals in Ho Chi Minh City and nearby provinces, such as Tu Du Hospital, Hung Vuong Hospital, Phuong Chau International Hospital, and the Hospital of Obstetrics and Gynecology Can Tho City, have applied GBS assessment between 35 and 37 weeks of gestation and used internal treatment regimens in which the prestige guidelines recommended by the above international associations are adopted. This is because GBS assessment during pregnancy, especially in the last three months, in combination with antibiotic prophylaxis at delivery as proposed, has been proven to eliminate early-onset neonatal sepsis, reduce the proportion of children with severe sequelae due to the effects of neonatal infection, and reduce infant mortality [13,18].

GBS diagnostic methods such as conventional PCR, qPCR, and culture are currently used [19]. The necessity to return diagnostic results in a shorter amount of time, mainly for pregnant women in labor whose GBS infection status has not yet been determined, is not addressed by traditional culture procedures, which take two to three days to provide diagnostic results. A PCR method, specifically qPCR, is adapted to receive the results of a GBS test faster. However, the time in which results are needed for a pregnant woman in labor is not met by existing qPCR techniques (GBS screening results must be delivered within 2 h of sampling). The only *in vitro* diagnostic test that entirely complies with CDC requirements for GBS screening during labor is GeneXpert (Cepheid) technology [20]. However, Vietnam does not have good access to this system. Here, we report two qPCR systems based on the *sip* and *cfb* genes, with previously designed primers and probes combined with optimized conditions, leading to high GBS diagnostic values. The study aimed to compare improved qPCR processes, which aid in the speedy detection of GBS infection in pregnant women in labor, with the culture method as the gold standard [19] to ascertain the diagnostic value of the procedures. Additionally, the frequency of GBS infection in pregnant women during labor was also identified.

## 2 Materials and methods

### 2.1 Study design

This descriptive and cross-sectional study collected patient demographic characteristics and clinical data. Two in-house real-time PCR assays, a commercial real-time PCR kit, and a chromogenic culture method, were compared and used to evaluate the prevalence of GBS colonization in pregnant women in the last trimester of pregnancy and at delivery.

### 2.2 Study population

The study population comprised pregnant women at 35 weeks gestation or greater who attended antenatal clinics at University Medical Center Ho Chi Minh City-Branch 2, Ho Chi Minh City, between April 2021 and February 2022. Women who had taken antibiotics used vaginal suppositories or performed vaginal douching within 48 h, and those with premature membrane rupture, premature birth, or age below 18 years were excluded from the study. Clinical indicators of early-onset neonatal infection followed the NICE guidelines [21].

### 2.3 Sample size

The sample size was calculated based on the prevalence rate of GBS colonization in pregnant women reported in a previous study, which was 8.02 % [22], at a 95 % confidence level and a margin of error set at 0.05. The estimated sample size for the prevalence study was 114 participants. The optimal sample size for estimating the sensitivity and specificity of the improved qPCR assays was determined, setting the same error probability and confidence level. The expected specificity and sensitivity were determined at 99 % for both. Considering the aforementioned estimated GBS prevalence, the minimum sample size was 200 for sensitivity and specificity. Ultimately, a total of 259 subjects were screened.

### 2.4 Clinical specimen collection and DNA extraction

The rectovaginal samples were collected by Amies Transport Medium without Charcoal swabs (Cat. 1530, Condalab) for the preservation of bacteria and transferred to the laboratory at 2–8 °C within an hour for GBS testing by chromogenic culture and real-time PCR assays [23]. The swabs were immersed in 2 ml of 1X PBS in 15 mL Falcon tubes and vortexed for at least 30 s. For the chromogenic culture method, two hundred microlitres of cell fluid were placed into Todd Hewitt Broth + Antibiotics proliferative medium (REF 42 116, Biomerieux). Another corresponding volume was used for DNA extraction, followed by real-time PCR testings. The total genomic DNA was extracted by the column-based DNA extraction kit (*AccuRive* viral sDNA/RNA prep kit, *ref. No.* EX-DRA03.1A, Khoa Thuong Biotech Co., Ltd.) precisely following the manufacturer's instructions. The total time for DNA extraction was approximately 30 min per sample. The DNA was eluted in 50 μL of the elution buffer provided in the kit and stored at 4 °C for instant use or at −20 °C until use. Five microlitres of the DNA solution were used for qPCR testing.

### 2.5 Chromogenic culture conditions and bacteria identification

The samples in proliferative media were incubated for 24 h at 37 °C in an aerobic atmosphere. After 24 h, 100 μL of proliferated bacterial fluid was cultured on chromID™ Strepto B agar (STRB) (REF 43 461, Biomerieux) at 37 °C, and the results were read after 24–48 h. Negative plates did not have pink colonies. Plates with pink colonies were retested with Latex Wellcogen Strep B (REF ZL20/R30858701, Oxoid Ltd.). If there was white precipitation, GBS infection was confirmed.

### 2.6 Real-time PCR assays

Primers and probes specific to GBS from previous studies [24,25] and those for self-designed internal controls (Table 1) were synthesized by Integrated DNA Technologies (IDT). For GBS, probes were labeled with a FAM fluorescent tag at the five prime ends and contained internal ZEN and Iowa Black fluorescent quenchers at the three prime ends. For the internal control (IC), probes were labeled with a HEX fluorescent tag at the 5 prime ends, and other components similar to probes were used for GBS detection.

**Table 1:** Primer and probe sequences for qPCR.

Name	Gene	Sequences (5’ – 3′)	Length (bp)	Products (bp)	Origin
F1	*cfb*	GGGAACAGATTATGAAAAAMCG	22	126	[24]
R1	*cfb*	AAGGCTTCTACACGACTACCAA	22		
P1	*cfb*	FAM-AGACTTCATTGCGTGCCAACCCTGAGAC-BHQ1	28		
F2	*sip*	GTTCCAGCAGCTAAAGAGGAAG	22	118	[25]
R2	*sip*	CCGGTGCTACTTTAGCTACTGG	22		
P2	*sip*	FAM-CACCAGCTTCTGTTGCCGCTGAAACACCAGC-BHQ1	31		
F3	RPLPO	GCAGATCCGCATGTCCCTTCGC	22	106	This study
R3	RPLPO	CTCCAGAGCTGGGTTGTTTTCCAGG	25		
P3	RPLPO	HEX-AGGCTGTGGTGCTGATGGGCAAGAACACCATGATG-BHQ1	35		

The real-time PCR assay targeting the *cfb* gene consisted of the F1/R1 primers (600 nM), the P1 probes (200 nM), the internal control F3/R3 primers (300 nM), the internal control P3 probe (100 nM), MgCl_2_ (4 mM), h-Taq DNA polymerase (1.5 units), dNTP mix (200 μM), 1X PCR buffer and 5 μL of the extracted DNA in a total reaction volume of 25 μL.

For the *sip* gene, the real-time PCR assay included the F2/R2 primers (200 nM), the P2 probes (100 nM), the internal control F3/R3 primers (100 nM), the internal control P3 probe (50 nM), MgCl_2_ (2 mM), h-Taq DNA polymerase (2 units), dNTP mix (200 μM), 1X PCR buffer and 5 μL of the extracted DNA in a total reaction volume of 20 μL.

The endpoint in the real-time PCR system is meticulously determined by detecting specific gene targets (*cfb* and *sip* genes in our study), indicating Group B Streptococcus (GBS) presence. A positive endpoint is characterized by amplifying and detecting these gene targets, while a negative endpoint indicates their absence. Fluorescence is precisely monitored during the amplification cycles for each real-time PCR assay. Based on the fluorescence signal, a threshold cycle (Ct) value is established to determine whether the sample is positive, negative, or sometimes indeterminate.

*Positive Endpoint:* A sample is considered positive if the fluorescence signal crosses a predefined threshold level after 40 cycles. This threshold is typically set above the background noise and indicates that the target sequence is present in the sample. The Ct value (Cycle threshold) is used to determine positivity. A lower Ct value indicates a higher concentration of target material, while a higher Ct value suggests a lower concentration.

*Negative Endpoint:* A sample is negative if the fluorescence does not reach the threshold level after 40 cycles, indicating that the target genetic material is absent or below the detection limit.

*Indeterminate or Inconclusive Endpoint*: Sometimes, if the signal is ambiguous or falls within a specific range that cannot conclusively determine the presence or absence of the target, the result may be labeled as indeterminate or inconclusive. This often requires retesting.

The optimal amplification of the target gene fragments was set at 95 °C for 15 min, followed by 40 cycles of denaturation at 95 °C for 15 s and annealing/extension at 60 °C for 20 s. The endpoint is determined using positive and negative controls during the PCR run. Positive controls include known strains of *S. agalactiae* ATCC® 12386™, ATCC® 13813™, and ATCC® 12401™, while the negative control uses a dilution buffer instead of a DNA template. The internal controls ensure that the real-time PCR reaction functions correctly. Our study meticulously evaluated different real-time PCR systems (e.g., Mx3005P, CFX96, AriaMx, and Roto-Gene Q) to ensure robust and consistent results, which were available within an hour for rapid detection of GBS.

### 2.7 Statistical analysis

Descriptive statistical analysis was used to describe the demographic and clinical characteristics of the women and their neonates, including age, techniques for childbirth, length of hospital stay, prenatal antibiotic administration, sex, weight, and early-onset neonatal infection status, and the kappa coefficient was evaluated using the Statistical Package for Social Science (SPSS) version 26.0. The performance characteristics, including the sensitivity, specificity, positive/negative predictive values, and accuracy of the two in-house real-time PCR assays and the commercial real-time PCR kit, were calculated by MedCalc software (http://www.medcalc.org/calc/diagnostic_test.php). The proportion of GBS colonization discovered in this study was compared to the one previously published using a one-sample binomial test to see if the values were equal. Significant results were defined as *p-values* of 0.05.

## 3 Results

### 3.1 Demographic and clinical characteristics

Overall, 259 qualified pregnant women were recruited in this study. Details on the demographic and clinical characteristics of these participants and their neonates are presented in Table 2. The mean age of the patients was 30.2 ± 5.0 years, ranging from 19 to 46 years. Age was distributed normally (*p* = 0.267). There were three types of deliveries, including the caesarean section (67.2 %), assisted vaginal delivery (1.0 %), and natural birth (31.8 %). The length of hospital stay ranged from 3 to 8 days, with the most significant proportion of 5 days (56.5 %), followed by 3 days (20.7 %) and then 4 days (18.1 %). Other lengths of hospital stay, for instance, 6, 7, and 8 days, covered smaller percentages of 2.1 %, 1.6 %, and 1.1 %, respectively. A gestational age at delivery equal to or greater than 37 weeks covered 96.6 % of the included patients. Half of the sample consisted of boys, while the other half consisted of girls. The highest percentages of neonatal weights were 42.9 % and 42.3 % for 3000–3500 and 2500–3000 g, respectively. 11.0 % of the neonates had a weight larger than 3500 g, and only a small percentage (3.8 %) weighed 2000–2500 g. Intrapartum antibiotic prophylaxis was provided for 34.7 % of the patients. Early-onset neonatal infection occurred at a rate of 4.7 % (Table 2).

**Table 2:** Patient demographic and clinical data.

Characteristics	Number (n)	Percentage (%)/Mean ± SD
Pregnant women		
Age (years)	258	30.2 ± 5.0
Methods of giving birth	198	
Natural birth	63	31.8
Assisted vaginal delivery	2	1.0
Caesarean section	133	67.2
Length of hospital stay (days)	193	
3	40	20.7
4	35	18.1
5	109	56.5
6	4	2.1
7	3	1.6
8	2	1.1
Intrapartum antibiotic prophylaxis	193	
Yes	67	34.7
No	126	65.3
GBS detection	259	
Culture	66	25.5
*cfb*-based real-time PCR	66	25.5
*sip*-based real-time PCR	64	24.7
Commercial real-time PCR kit	41/187	21.9
Neonates		
Sex	178	
Male	89	50.0
Female	89	50.0
Age (weeks)	149	38.4 ± 0.8
<37	5	3.4
≥37	144	96.6
Weight (grams)	182	3105 ± 335
2000-2500	7	3.8
2500-3000	77	42.3
3000-3500	78	42.9
>3500	20	11.0
Early-onset neonatal infection	193	
Yes	9	4.7
No	184	95.3

### 3.2 GBS detection

66 patients were determined to be GBS-positive by culture, and 66 and 64 patients were determined to be GBS-positive by real-time PCR assays targeting the *cfb* and *sip* genes, respectively (Table 2). Overall, positive specimen proportions of 24.7 % and 25.5 % were detected by real-time PCR assays using the *sip* and *cfb* genes, respectively, compared to the culture method (25.5 %).

There were 21 samples where discrepancies arose among the four diagnostic techniques. To address nonspecific amplification, samples that yielded positive results from any real-time PCR assays under evaluation underwent sequencing. The most notable subset, consisting of 8 cases, exhibited positivity in both cultures and the two in-house real-time PCR assays yet tested negative with the commercial real-time PCR kit. Two smaller subsets emerged: one comprising 3 cases that tested positive with the commercial real-time PCR kit and another containing 3 cases that tested positive exclusively with the culture method but negative with the remaining techniques. Each residual group included a case showcasing divergent GBS testing outcomes across different methodologies (Table 3).

**Table 3:** Results of inconsistencies between the four assessed procedures.

Number of discordant samples (n)	Chromogenic culture	*cfb*-based real-time PCR	*sip*-based real-time PCR	Commercial real-time PCR
3	+	–	–	–
1	+	–	–	+
1	+	+	–	+
8	+	+	+	–
1	–	+	–	–
1	–	+	+	–
1	–	+	–	+
1	–	+	+	+
3	–	–	–	+
1	–	–	+	+

The positive and negative results are indicated with (+) and (−) signs, respectively.

### 3.3 The clinical values of real-time PCR assays

We applied the real-time PCR assays and culture method to rectovaginal swabs collected from pregnant women. We compared them to the criteria: “The samples confirmed as positive were identified as such by both in-house real-time PCR tests even if they were negative according to culture or by culture alone regardless of whether any of the real-time PCR assays indicated positivity or negativity.” The results are shown in Table 4. Each diagnostic method shows variations in sensitivity and specificity. The chromogenic culture method appears more sensitive than the commercial real-time PCR kit. Additionally, discrepancies exist among the real-time PCR assays and chromogenic culture, indicating the need for careful interpretation and possibly complementary use of multiple diagnostic methods.

**Table 4:** Diagnostic results of the four assessed procedures performed on rectovaginal specimens.

Diagnostic results		Criteria	
		Positive	Negative
*cfb*-based real-time PCR	Positive	64	2
	Negative	4	189
*sip*-based real-time PCR	Positive	63	1
	Negative	5	190
Commercial real-time PCR kit	Positive	36	5
	Negative	11	135
Chromogenic culture	Positive	66	0
	Negative	2	191

The data allow for comparing the efficacy of different methods in detecting GBS infection. The chromogenic culture method shows the highest sensitivity (97.1 %), specificity (100 %), PPV (100 %), NPV (98.9 %), and accuracy (99.2 %) among the methods listed. This indicates that the chromogenic culture method effectively correctly identifies positive and negative cases.

The in-house real-time PCR assays showed excellent clinical diagnostic values compared to the commercial real-time PCR kit. The accuracies were 97.6 % for both *cfb*-based and *sip*-based real-time PCR assays. The sensitivity, specificity, positive predictive value, and negative predictive value of the *cfb*-based and *sip*-based qPCR procedures were 94.1/92.7, 99.0/99.5, 97.1/98.5, and 97.8/97.3, respectively, while the corresponding values of the commercial real-time PCR kit were 76.6, 96.4, 89.0, and 91.6. The Kappa values for the two in-house real-time PCR assays were also higher than that of the commercial real-time PCR kit (0.940/0.939 vs. 0.763) (Table 5).

**Table 5:** Diagnostic values of the four corresponding procedures.

Values	*cfb*-based qPCR	*sip*-based qPCR	Commercial qPCR	Culture
Pearson's R	0.940 [0.889–0.982]	0.940 [0.890–0.980]	0.766 [0.643–0.868]	0.980 [0.949–1.000]
Kappa	0.940 [0.888–0.981]	0.939 [0.887–0.980]	0.763 [0.635–0.867]	0.980 [0.947–1.000]
Sensitivity	94.1 [85.6–98.4]	92.7 [83.7–97.6]	76.6 [62.0–87.7]	97.1 [89.8–99.6]
Specificity	99.0 [96.3–99.9]	99.5 [97.1–100]	96.4 [91.9–98.8]	100 [98.1–100]
PPV	97.1 [89.5–99.3]	98.5 [90.4–99.8]	89.0 [77.1–95.1]	100 [94.6–100]
NPV	97.8 [94.5–99.1]	97.3 [93.9–98.8]	91.6 [86.7–94.8]	98.9 [95.8–99.7]
Accuracy	97.6 [95.0–99.1]	97.6 [94.9–99.1]	91.0 [85.9–94.7]	99.2 [97.2–99.9]

Disease prevalence: 27.4 %; PPV: Positive predictive value; NPV: Negative predictive value.

## 4 Discussion

In this study, we evaluated the characteristics and applications of the real-time PCR assays and the chromogenic culture method to ascertain the prevalence of GBS colonization in Vietnamese pregnant women. The exploration of real-time PCR assays as diagnostic tools was an objective in numerous reports. Nevertheless, there have yet to be any similar studies conducted in Vietnam until now. In addition, the performance of a commercially available real-time PCR kit for detecting GBS was also assessed.

The prevalence of GBS (25.5 %) detected by the *cfb*-based real-time PCR assay and chromogenic culture method in this study was statistically higher than that in other studies, such as that by Kate et al. (19.5 %; *p* = 0.011) in Jordan [26] and Lakshmi et al. (12.9 %; *p* < 0.001) in India [27], and much higher than the finding recently published by Vu et al. (8.02 %; *p* < 0.001) at a hospital in Hanoi, Vietnam, from 2016 to 2020 [22]. However, the GBS prevalence in this study agreed with that reported by Carrillo-Avila et al. (27.81 %; *p* = 0.223) in Spain [28] and by Fernanda et al. (26.99 %. *p* = 0.320) in Brazil [29].

Various factors could account for the disparity in the GBS-positive cases among studies, including population ethnicity, geographic location, demographics, medication use, sample size, study duration, and screening methods [7,30]. The study found statistically significant differences in GBS colonization incidence across detection methods (*p* < 0.05) (Table 2). All three PCR assays and the chromogenic culture method revealed a high GBS prevalence of up to 28.2 % [30]. These findings highlight the significant risk of GBS infection and its potential to cause neonatal sepsis in southern Vietnam.

We additionally assessed the discrepancies among the GBS screening methods employed in the study (Table 3). These variations could stem from clinical specimens collected with low bacterial counts or nonviable bacteria. The diagnostic efficacy of each method was determined based on significant features observed in previous studies [7,28,30,31]. Contradictory results were resolved using gene sequence analysis for positive samples only with real-time PCR assays [28]. The sequenced PCR products demonstrated the accurate amplification of *cfb* or *sip* genes from GBS. However, some samples failed to yield PCR products primarily due to the low concentration of the target pathogen (data not disclosed).

Furthermore, studies have shown that real-time PCR aids in diagnosing GBS colonization in patients whose swabs tested negative using the culture approach for verification [28]. Potential explanations include inadequate preservation of rectovaginal swabs during collection and transportation, resulting in nonviable bacteria that do not grow in the proliferative medium utilized [32], insufficient bacterial concentrations, likely falling below the detection limit of the chromogenic culture method [32,33], An elevated density of rectovaginal flora, pregnant women receiving antimicrobial chemotherapy before testing, or employing feminine hygiene products that could impede GBS growth, even on selective medium [20,29].

Technical issues, such as PCR inhibition, genetic variations, or cross-contamination, may explain why some samples were positive by culture but negative by PCR. The direct real-time PCR protocol, used without bacterial enrichment per CDC guidelines and certain studies [19,31], provided results within 2 h. The rationale behind this decision was to expedite the delivery of diagnostic results for patients who lacked information about their GBS colonization status at the time of delivery. By implementing this approach, 18 % of GBS-positive specimens identified by culture but not by direct real-time PCR have been reported [31]. In this study, 6.1 %–7.6 % of samples were culture-positive but PCR-negative, likely due to low-titre samples. This suggests that enrichment before PCR testing may be needed in some cases [19].

Even though direct real-time PCR assays demonstrate lower sensitivity (<75 %) compared to the gold standard based on prior research [34]. The two direct real-time PCR assays assessed in our study presented high sensitivity (91.4–93.9 %) and specificity (97.9–100 %) compared to the culture method with chromogenic medium (data not shown). The results might also contain no exceptions for the previously described false-positive chromogenic agar results, and we suggested that further testing be carried out to ensure that only GBS is found and no other *Streptococcus* spp. [35], even though it was developed and improved to select for GBS colonies, including nonhemolytic/non-pigmented isolates [31]. The values of the two direct real-time PCR assays were even better than the criteria (Table 5).

The *cfb*-based and *sip*-based real-time PCR assays exhibit high sensitivity and specificity, making them highly effective in detecting actual GBS-positive cases with minimal false positives. In contrast, the commercial assay shows lower sensitivity, increasing the likelihood of missed GBS cases and making it less reliable for clinical decision-making when rapid and accurate results are crucial. Both in-house assays also outperform the commercial assay regarding PPV, NPV, and overall accuracy, with excellent agreement with the reference standard, while the commercial assay demonstrates only substantial agreement. This further highlights the better performance and reliability of the in-house assays. Overall, the *cfb*-based and *sip*-based assays, nearly equivalent in their diagnostic performance, are more reliable for rapid and accurate GBS screening, making them particularly suitable in a clinical setting where timely detection is needed to guide antibiotic prophylaxis during labor.

Although the culture method has slightly higher sensitivity and perfect specificity, the *cfb*-based and *sip*-based qPCR assays perform very closely, with minimal differences in specificity, accuracy, and Kappa values, indicating a high ability to identify actual positive GBS cases correctly. These minor differences are not clinically significant. They offer a practical advantage because qPCR assays provide results much faster (within 2 h compared to 48–72 h for culture). In conclusion, while culture is marginally superior, qPCR assays are highly competitive and offer a rapid, nearly equivalent diagnostic value for detecting GBS.

Notably, the chromogenic culture method demonstrates the highest Pearson's correlation and Kappa values, signifying robust correlation and consensus with the criteria, but results take 48–72 h. In contrast, the real-time PCR techniques provide results within 2 h and demonstrate high sensitivity, specificity, PPV, NPV, and accuracy. The commercial PCR kit still shows respectable diagnostic values despite slightly lower performance. The data suggests that while all real-time PCR methods effectively diagnose GBS infection, the chromogenic culture method is the most reliable in some instances. Clinicians may opt for different PCR methods based on cost, speed, accuracy, and technical expertise. Each diagnostic method has limitations, such as cost and time, which should be considered. Depending on the clinical context, these results can guide clinicians in choosing the most effective method for diagnosing GBS infection.

The sampling technique is one of the crucial factors affecting the precision of diagnostic methods [20,36]. Discrepancies may have arisen due to the multiple swabs for each test [31]. Initially, we utilized two swab samples, the first for microbiological culture and the second for real-time PCR testing, as previously described [28]. Nevertheless, there were significant differences in the diagnostic results between the methods (data not shown). Ultimately, we employed a single swab sample for all GBS screening tests to mitigate discrepant results. Repeating the diagnostic procedures (using the previous protocols or a completely new protocol with comparable diagnostic capabilities) may be the most appropriate action in conflicting results. Through repeated testing, clinicians can make the most precise assessment of GBS infection in pregnant women, thus enabling appropriate treatment decisions.

To evaluate the values of the techniques used in the study, we used the criteria [28] as a gold standard, the criterion defined positive samples as those identified by both PCR assays (even if the culture was negative) or by culture alone. As depicted in Table 5, the two *cfb*-based and *sip*-based real-time PCR assays exhibited excellent diagnostic efficacy, surpassing the commercial real-time PCR kit and matching the culture method, making them suitable for GBS testing. Although the culture method demonstrated the highest values, the in-house PCR assays performed similarly. The commercial kit's lower performance could be due to variations in bacterial strains and gene targets, warranting further evaluation in future studies.

The sensitivity (92.7–94.1 %) and specificity (99.0–99.5 %) of the in-house real-time PCR assays were comparable to those (84.4–98.5 % and 94.6–99.6 %, respectively) of commercial diagnostic assays, such as the GeneXpert kit (Cepheid, Sunnyvale, CA, USA), and other published real-time PCR assays for intrapartum Group B streptococcus screening and the prevention of neonatal sepsis [20,28,37,38]. Due to their high sensitivity and specificity and shorter turnaround times compared to chromogenic culture, the two real-time PCR assays investigated in this study are suitable for GBS screening procedures. Furthermore, they could contribute to reducing the cost per test, rendering them economically viable as a screening method at obstetrical centers to mitigate newborn illnesses caused by GBS in developing countries like Vietnam. In addition, we did not encounter any invalid results with the Xpert® GBS PCR in 8.2–13.4 % of the cases, as reported in previous studies [33,36].

Our study encountered several limitations. Initially, we only collected samples from a subset of the enrolled women at delivery time. Consequently, we could not determine which women benefited from our real-time PCR assays regarding time-to-results and cost per test. Secondly, according to a previous study, certain discrepancies between the real-time PCR results and the GBS culture could be attributed to repeated sampling at various stages during pregnancy [20]Thirdly, some clinical data were omitted due to specific clinical circumstances. To address this limitation, it is imperative to acquire comprehensive data on clinical and demographic characteristics.

Nonetheless, a notable strength of our study is that it represents one of the few investigations on GBS and the first study to evaluate real-time PCR assays and the chromogenic culture method used for GBS detection in Vietnam. Our findings offer compelling evidence supporting the use of direct real-time PCR assays for the reliable and expeditious detection of 10.13039/100019980GBS colonization in pregnant women, underscoring its high clinical utility. This study is poised to alleviate clinicians' apprehensions regarding the efficacy of direct real-time PCR assays in assessing GBS colonization among pregnant women in Vietnam.

## 5 Conclusions

Our study demonstrated that direct real-time PCR assays and a chromogenic culture approach for GBS screening yielded highly consistent results using correct sampling techniques. The real-time PCR assays targeting specific *cfb* and *sip* genes proved effective for GBS screening, exhibiting excellent diagnostic accuracy compared to the predefined criteria as reference standards. Moreover, direct real-time PCR assays offered short turnaround times, particularly for pregnant women in labor.

## CRediT authorship contribution statement

**Manh-Tuan Ha:** Writing – original draft, Supervision, Resources, Project administration, Methodology, Funding acquisition, Conceptualization. **Huyen Tran-Thi-Bich:** Investigation. **Thao Bui-Thi-Kim:** Investigation. **My-Linh Nguyen-Thi:** Visualization, Software, Formal analysis, Data curation. **Thanh Vu-Tri:** Writing – review & editing, Resources, Investigation, Funding acquisition. **Thuy-Duong Ho-Huynh:** Writing – review & editing, Validation, Supervision, Resources. **Tuan-Anh Nguyen:** Writing – review & editing, Writing – original draft, Visualization, Validation, Software, Project administration, Methodology, Funding acquisition, Formal analysis, Data curation, Conceptualization.

## Ethics approval and consent to participate

The ethics committee approved the study of the University of Medicine and Pharmacy at Ho Chi Minh City, Vietnam (reference number: 370/HĐĐĐ-ĐHYD, June 02, 2020). All participants provided written informed consent. This work was carried out following the Code of Ethics of the World Medical Association (Declaration of Helsinki) for Experiments in Humans.

## Consent for publication

Not applicable.

## Availability of data and materials

The detailed data and materials are available upon reasonable request (anh.nt@umc.edu.vn).

## Competing interests

The authors declare that they have no competing interests. All the authors have read and approved the final version of the manuscripts.

## Funding

This study was funded by a research grant (reference number: 32/2020/HĐ-QPTKHCN, June 25, 2020) from the 10.13039/100021014Department of Science and Technology Ho Chi Minh City (DOST HCMC) in Vietnam.

## Declaration of competing interest

The authors declare that they have no known competing financial interests or personal relationships that could have appeared to influence the work reported in this paper.

## Data Availability

Data will be made available on request.
